# Bioabsorption and Bioaccumulation of Cadmium in the Straw and Grain of Maize (*Zea mays* L.) in Growing Soils Contaminated with Cadmium in Different Environment

**DOI:** 10.3390/ijerph14111399

**Published:** 2017-11-16

**Authors:** Jorge Retamal-Salgado, Juan Hirzel, Ingrid Walter, Iván Matus

**Affiliations:** 1 Faculty of Agronomy, Universidad de Concepción, Vicente Méndez 595, Casilla 537, Chillán 3812120, Chile; jorgeretamal@unach.cl; 2 Faculty of Engineering and Business, Universidad Adventista de Chile, km 12 Camino a Tanilvoro, Chillán 3780000, Chile; 3 Instituto de Investigaciones Agropecuarias INIA, Avenida Vicente Méndez 515, Chillán 3800062, Chile; imatus@inia.cl; 4 Instituto Nacional de Investigación y Tecnología Agraria y Alimentaria (INIA), Apdo. Correos 8111, Madrid 28080, Spain; inwal@hotmail.com

**Keywords:** bioabsorption, heavy metals, translocation factor, tolerance index, food chain contamination

## Abstract

There is a worldwide increase of heavy metal or potentially toxic element (PTE), contamination in agricultural soils caused mainly by human and industrial action, which leads to food contamination in crops such as in maize. Cadmium (Cd) is a PTE often found in soils and it is ingested through food. It is necessary to determine the bioabsorption, distribution, and accumulation levels in maize to reduce or prevent food chain contamination. Cadmium absorption and accumulation in three maize cultivars were evaluated in three agricultural environments in Chile by increasing CdCl_2_ rates (0, 1, and 2 mg·kg^−1^). Evaluation included Cd accumulation and distribution in different plant tissues, bioaccumulation factor (BAF), bioconcentration factor (BCF), translocation factor (TF), and tolerance index (TI). Cadmium whole-plant uptake was only affected by the CdCl_2_ rate; the highest uptake was obtained with 2 mg·kg^−1^ CdCl_2_ (34.4 g·ha^−1^) (*p* < 0.05). Cadmium distribution in the maize plant usually exhibited the highest accumulation in the straw (*p* < 0.05), independently of the environment, Cd rate, and evaluated cultivar. Given the results for TF (TF > 2) and BAF (BAF > 1), the Los Tilos and Chillán environments were classified as having a high capacity to contaminate the food chain for all evaluated cultivars.

## 1. Introduction

There is a worldwide increase of potentially toxic element (PTE) contamination in agricultural soils caused mainly by human and industrial action, which has generated concern for food contamination by these metals [1]. PTEs present in soils cannot be degraded; cadmium (Cd), of these PTEs, is usually found in different soil types [2]. Cadmium is not an essential plant nutrient; however, this metal can be absorbed in greater amounts than other elements without any adverse effects on growth [3]. It interacts with the metabolism of other essential metals, such as calcium, zinc, and iron [4,5,6] and can be bioaccumulated and/or biotransformed by plants [3]. 

Cadmium intake through the food chain can become toxic for living organisms and is carcinogenic for human beings [7]. Cadmium can be absorbed in human beings through food, especially leaves and grains; it accumulates in the liver and kidneys for more than 30 years and causes health problems [7,8]. Toxicity of this metal involves kidney and skeletal organs and is largely the result of interactions between Cd and essential metals, such as calcium [8]). A daily intake of 1 mg·kg^−1^ body weight is considered toxic for human beings [9]. On the other hand, the soil Cd concentration considered to pose a risk is 1.0 mg·kg^−1^ [10,11]. 

Crops and cultivars usually differ in their capacity to absorb, accumulate, and tolerate Cd [12,13]. Among the agricultural crops that are important in the human diet, the main species that absorb and translocate Cd to the grain are hard wheat (*Triticum turgidum* L. var. *durum*), maize (*Zea mays* L.), wheat (*Triticum aestivum* L.), oat (*Avena sativa* L.), and rice (*Oryza sativa* L.). These have exhibited Cd concentrations over the maximum permitted for human health [14,15,16,17]. The amount of Cd in the soil depends mainly on physical and chemical properties, such as fertilization with phosphorus (P) and nitrogen (N), organic amendment applications, and exposure to sources of contamination [1,12,18,19]. 

Among the important agricultural crops, more specifically cereals, maize is revealed to be the second most important for cultivated area worldwide. Given an increasing demand for food, a larger land area accessible for agricultural use will be necessary in which the total available Cd concentration is not over the critical 1 mg·kg^−1^ level [20,21]. At the same time, a tendency for high Cd concentrations in the grain has been observed in new cereal crop cultivars worldwide. According to Cd distribution in the different plant organs, more than 40% of Cd is absorbed and translocated to the aerial part of the plant (grain and straw), and it could be directly (grains) or indirectly (animals) ingested and negatively affect humans [22,23,24]. Therefore, soils with higher Cd concentrations associated with the cultivation of cultivars with higher Cd accumulation capacity in the aerial part will generate a higher risk of ingesting this metal. To reduce food chain contamination, the present study evaluated plant Cd absorption, distribution, or translocation, and accumulation in different maize cultivars cultivated in different environments and with increasing soil Cd rate treatments.

## 2. Materials and Methods 

### 2.1. Climatic and Soil Characteristics of Each Environment

Three field experiments were conducted during the 2013–2014 season in different agricultural environments in Chile. The three environments and their characteristics were as follows: La Serena (30°3’ S; 71°14’ W) has soil of alluvial-colluvial origin (Typic Haplocambids) [25], an arid climate with a maritime influence, and had 40 mm precipitation concentrated during the winter. Los Tilos (33°34’ S; 70°37’ W) has soil of alluvial origin (Haploxeroll) [25], a semi-arid and temperate Mediterranean climate with hot and dry summers and cold winters, and had 163 mm precipitation. Chillán (36°31’ S; 71°54’ W) has soil of volcanic origin (Melanoxerand) [25], temperate Mediterranean climate with hot and dry summers and cold and humid winters, and reached 672 mm precipitation concentrated during the winter and the beginning of spring [26]. 

The physical and chemical properties of each soil are shown in Table 1. Soil samples were collected at two depths (0–0.2 and 0.2–0.4 m) and the physical and chemical properties of each soil analyzed at the start of the experiment are displayed in Table 1; analyses were performed according to the methodology indicated by Sadzawka et al. [27]. Soil hydrogen potential (pH) was measured in a ratio of 1:2.5 soil:water solution with a pH meter. Soil organic matter (OM) was measured by the Walkley–Negro wet digestion method. Available soil N (NO_3_-N and NH_4_-N) was extracted with 1 M KCl_2_ and was determined by colorimetry in a Skalar autoanalyzer (segmented flow spectrophotometer). Available P in the soil sample was determined by 0.5 M NaHCO_3_ (Olsen P) using the ascorbic acid molybdate method. Exchangeable calcium (Ca), magnesium (Mg), potassium (K), and sodium (Na) were determined by extraction of 1 M Ac. NH_4_O followed by flame spectroscopy: absorption (Ca and Mg) and emission (K and Na). The concentration of exchangeable soil aluminum (Al) was determined through extraction of 1 M KCl by absorption spectroscopy. Soil concentrations of iron (Fe), manganese (Mn), zinc (Zn), and copper (Cu) were determined in the diethylenetriamine pentaacetate, (DTPA) extract [28] by atomic absorption spectrometry (AAS). Boron (B) was determined by colorimetry in a solution obtained by acid digestion. Total soil and plant Cd was determined by electro-thermal atomic absorption spectrophotometry (graphite furnace technique) with Thermo Elemental Solar M5 equipment coupled to a model GF95 graphite furnace. Samples were digested in a microwave oven (MARS-Xpress, CEM Corporation, Matthews, NC, USA) before the spectrophotometry readings. For each soil sample, 0.5 g was weighed and 10 mL nitric acid 65% (Suprapur, Merck, Darmstadt, Germany) was added. For the plant tissue samples, 1 g dry matter (DM) maize was placed in a digestion tube and 10 mL Suprapur HNO_3_, +1 mL 30% H_2_O_2_ was added. Quality control for the analyses was based on certified reference material (International Plant-analytical Exchange Programme 979 for plant tissue and International Soil-analytical Exchange Programme 981 for soil), sample comparison between laboratories, internal control samples, and duplicates [29]. 

### 2.2. Cadmium Rates and Maize Genotypes

Cadmium was applied as CdCl_2_ (61.3% Cd) and the rate of this salt was 0 (Control), 1, and 2 mg·kg^−1^ adjusted for a 0–0.2 soil depth taking into account the apparent density of each soil (Table 1). The equivalent amount of Cd applied at the 1 and 2 mg·kg^−1^ rates of CdCl_2_ in each environment was: La Serena 2157.76 and 4315.52 g·ha^−1^, respectively; Los Tilos 1593.8 and 3187.6 g·ha^−1^, respectively; and Chillán 1226.0 and 2452.0 g·ha^−1^, respectively.

The maize cultivars used in the present study were Syngenta NK 703, Pionner P32D12, and Dekalb DK 627 in La Serena and Los Tilos and Syngenta NK exp, Pionner P 37W05, and Dekalb DK 469 in Chillán. These selected Syngenta, Pionner, and Dekalb cultivars have different genetic characteristics because companies have different genetic lines, which allows assessing the cultivar on the parameters to be evaluated in the plant.

### 2.3. Agronomic Management of the Experiment

Agronomic management practices were standardized for all the locations. The fertilization rates of N, P, and K were 360, 120, and 120 kg·ha^−1^, respectively, and the sources of fertilization were urea, triple superphosphate, and potassium chloride. Nitrogen was applied 30% and 70% at sowing and at the six-leaf stage, respectively. Both P and K were applied 100% at sowing.

Each experimental unit consisted of six rows 3 m long with 0.6 m row spacing (10.8 m^2^). The cultivated area in each environment was 291.6 m^2^ for three Cd rates, three maize cultivars, and three replicates. Experiments were sown on 8, 17, and 24 October 2013 in La Serena, Los Tilos, and Chillán, respectively, and the sowing rate was eight seeds per linear meter. The seedbed in each environment was prepared with a plow at 0.3 m depth followed by a surface cultivator.

Seven irrigation events were applied to all the environments, the first after sowing until the milk stage to complement accumulated precipitation between July and December 2013 (8.7, 44.5, and 334.7 mm in La Serena, Los Tilos, and Chillán, respectively) (Table 2). Each irrigation event was 50–60 mm, which was enough to maintain the soil with adequate moisture during crop development. The pre-emergent herbicide, a mixture of atrazine and S-metolachlor, was applied at a rate of 6.0 L·ha^−1^ at the post-emergence stage to control dicotyledonous weeds. The insecticide chlorpyrifos was applied before sowing at a rate of 5.0 L·ha^−1^ to control larvae in the soil. Diseases and incidence of insects during crop growth were very low in the three environments; therefore, foliar fungicides or insecticides were not used. Plots were harvested on 22, 24, and 29 April 2014 in La Serena, Los Tilos, and Chillán, respectively.

### 2.4. Plant Tissue Dry Matter, Soil Analysis, and Plant Tissue

The crop was harvested when the grains reached 15% moisture content. Plant samples with roots were collected from 1.0 m^2^ in each experimental unit and DM production was determined in the grain, straw (stem + leaf), and root. Tissue samples were washed with distilled water and oven-dried at 70 °C for 72 h to determine DM. At the end of the experiment, 10 soil sub-samples were collected from each plot at two depths (0–0.2 and 0.2–0.4 m); they were air-dried, later ground, and passed through a 2 mm sieve to determine total Cd concentration. The methodologies to determine Cd concentration in plant tissue and soil are mentioned above.

### 2.5. Data Analysis

The following factors were determined. 

#### 2.5.1. Bioconcentration and Bioaccumulation Factor 

The soil and plant Cd concentrations were calculated based on dry weight. The bioconcentration factor (BCF) and bioaccumulation factor (BAF) are indices of the plant’s capacity to accumulate Cd with respect to the concentration of the soil substrate. They are calculated as follows [30,31,32]:

BAF_grain_ = C_grain_/C_soil_ (1)

BAF_straw_ = C_straw_/C_soil_ (2)

BCF_root_ = C_root_/C_soil_ (3)

where C_grain_, C_straw_, and C_root_ are the Cd concentrations in the grain, straw, and root, respectively, and C_soil_ is the concentration in the soil.

#### 2.5.2. Translocation Factor

The translocation factor (TF) or mobilization ratio determines the relative displacement of Cd from the concentration of the plant part in the soil (root) toward the aerial parts of the plant (grain and straw). It is calculated by the following equations [33]:

TF_grain_ = C_grain_/C_root_ (4)

TF_straw_ = C_straw_/C_root_ (5)

where C_grain_, C_straw_, and C_root_ are the Cd concentrations in the grain, straw, and root, respectively.

#### 2.5.3. Tolerance Index

The tolerance index (TI), or the tolerance of plants to Cd, can be measured as the variation of the biomass response of the plant part (grain, straw, or root) by the toxicity of Cd compared with the biomass of the treatment with no added Cd. This index was determined by the following equation [34]:

TI = DM_Cd treatment_/DM_control_ (6)

where DM_Cd treatment_ and DM_control_ are the DM of each treatment with added Cd and DM of the control treatment, respectively.

### 2.6. Experimental Design and Statistical Analysis

The experimental design was a split sub-sub-plot where the main plot was the environment (3), the split plots were the Cd rates (3), and the split sub-sub-plots were the maize cultivars (3) with three replicates. Results were analyzed by ANOVA and Tukey’s test (*p* = 0.05) by the SAS PROC MIXED Model procedure (SAS Institute, Cary, NC, USA) and considering the locations as random effects. For the significant interactions, contrast analysis was used to compare the treatment effects separately.

## 3. Results

The environment and its interaction with the cultivar significantly affected DM production in the straw (Table 3). Dry matter production in the straw fluctuated between 7.0 and 14.7 Mg·ha^−1^ (Table 4). The highest value in all the cultivars was attained in La Serena (*p* < 0.05) then Chillán and Los Tilos; there were non-significant differences between them (*p* > 0.05) and values were 14.7, 9.3, and 7.0 Mg·ha^−1^, respectively (Table 4). Comparing DM production in the straw between cultivars, and as a mean of the environments and Cd rates, higher production was observed in Syngenta with 10.9 Mg·ha^−1^, followed by Pioneer and Dekalb, with values of 9.0 and 9.7 Mg·ha^−1^, respectively, and no differences between them (*p* > 0.05) (Table 4). 

Dry matter production in the roots was also affected by the environment, cultivar, and environment × cultivar interaction (Table 3). When comparing environments, the highest DM production in roots was found in La Serena (mean 2.0 Mg·ha^−1^), significantly higher than in Chillán (mean 1.3 Mg·ha^−1^) (*p* < 0.05), which, in turn, was significantly higher than in Los Tilos (mean 0.8 Mg·ha^−1^) (*p* < 0.05). The comparison between cultivars indicated that the highest DM production in roots was obtained in Syngenta (mean 1.5 Mg·ha^−1^), significantly higher (*p* < 0.05) than in Dekalb and Pioneer (mean 1.2 and 1.1 Mg·ha^−1^, respectively), and with non-significant differences between them (*p* > 0.05) (Table 4).

Cadmium uptake in the grain was affected by the environment, Cd rate, environment × Cd rate interaction, and environment × cultivar interaction (Table 3). When contrasting environments (Table 5), the highest Cd uptake in the grain was obtained in La Serena with a mean value of 0.45 g ha^−1^, which was significantly higher (*p* < 0.05) than in Chillán (0.12 g·ha^−1^) and Los Tilos (0.08 g·ha^−1^), and there were no differences between them (*p* > 0.05). The contrast between Cd rates (Table 5) indicated that the highest Cd uptake was attained with 2 mg·kg^−1^ CdCl_2_ (0.25 g·ha^−1^), which was significantly higher (*p* < 0.05) than for 1 mg·kg^−1^ CdCl_2_ (0.19 g·ha^−1^); the latter was significantly higher (*p* < 0.05) than the control (0.06 g·ha^−1^) (Table 5). The contrast between cultivars (Table 5) indicated that the highest Cd uptake in the grain was reached in Pioneer (0.19 g·ha^−1^), then in Syngenta (0.16 g·ha^−1^) and Dekalb (0.14 g·ha^−1^) with non-significant differences between them (*p* > 0.05) (Table 5). The environment × Cd rate and the environment × cultivar interactions exhibited greater variability associated with the environment and revealed high values obtained in La Serena (data not shown). 

Cadmium uptake in the straw was only affected by the Cd rate (Table 5). When contrasting the CdCl_2_ rates (Table 5), the highest Cd uptake in the straw was attained in Los Tilos with a mean of 23.9 g·ha^−1^ (*p* < 0.05), while La Serena and Chillán had 18.8 and 18.5 g·ha^−1^, respectively, and with a non-significant difference between them (*p* > 0.05).

As for Cd uptake in the roots, there was an effect of the environment and Cd rate with an interaction between both sources of variation and between environment × cultivar (Table 5). The contrast between environments (Table 5) indicated that the highest Cd uptake in the roots was obtained in La Serena with a mean of 4.5 g·ha^−1^, which was significantly higher than in Chillán and Los Tilos where uptakes were 0.6 and 0.2 g·ha^−1^, respectively; there was non-significant difference between them (*p* > 0.05). When contrasting the Cd rate (Table 5), higher Cd uptake in the roots was observed when using 2 and 1 mg·kg^−1^ CdCl_2_; however, they were both similar (*p* > 0.05) and values were 2.1 and 1.5 g·ha^−1^, respectively. They significantly surpassed the control (*p* < 0.05), which had an uptake of 0.6 g·ha^−1^.

**Figure 1:**
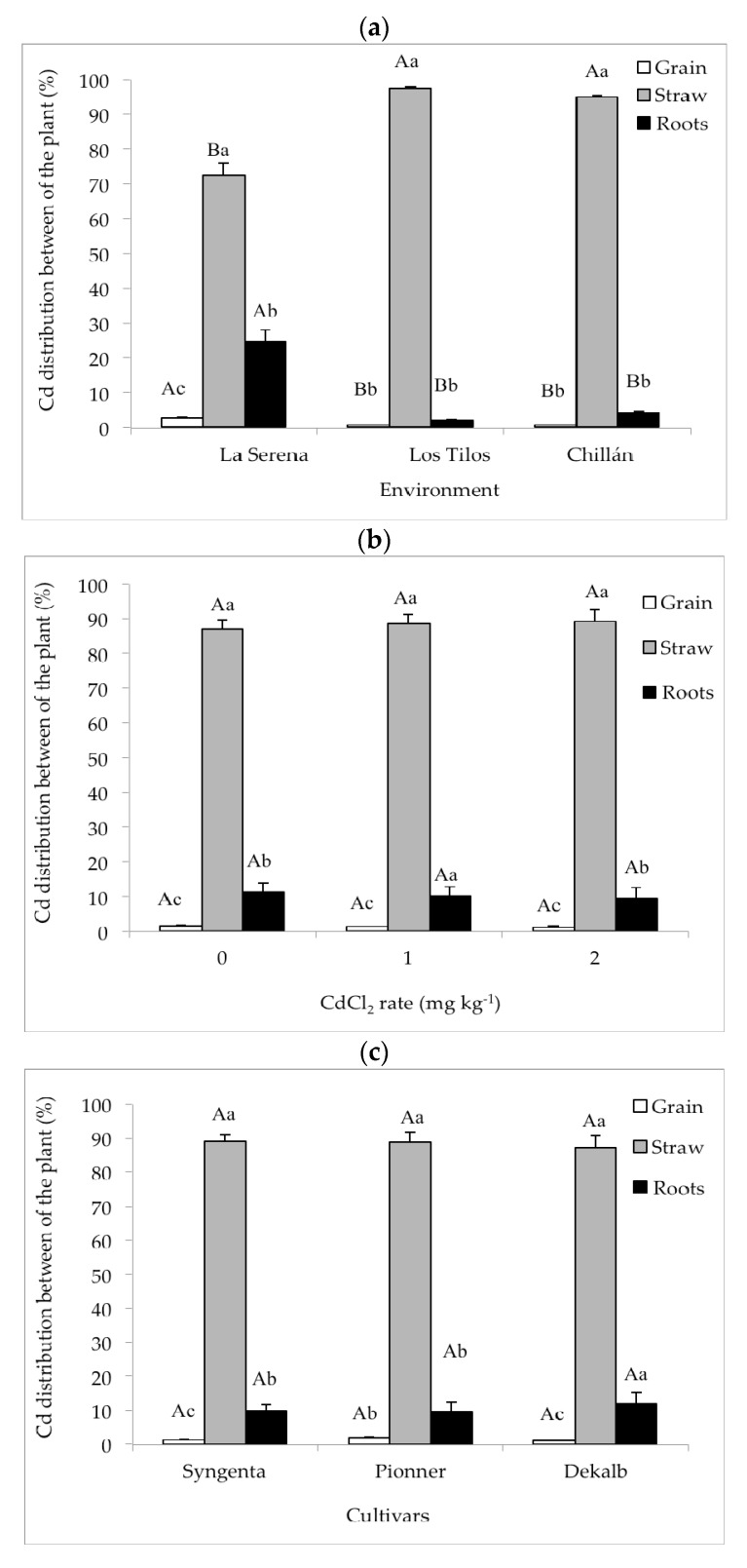
Cadmium distribution in the maize plant for different: (**a**) environments; (**b**) cadmium rates; and (**c**) maize cultivars.

Whole plant Cd uptake was only affected by the Cd rate (Table 3; Figure 1). The highest whole plant Cd uptake was attained with 2 mg·kg^−1^ CdCl_2_ (34.4 g·ha^−1^), which was significantly higher (*p* < 0.05) than with 1 mg·kg^−1^ CdCl_2_ (17.7 g·ha^−1^); the latter was significantly higher than in the control without Cd (2.9 g·ha^−1^) (*p* < 0.05) (Figure 1b). Although the cultivars did not show any differences in whole plant Cd extraction (Table 3, Figure 1c), these values were 21.5, 17.1, and 16.4 g·ha^−1^ in Dekalb, Pioneer, and Syngenta, respectively. Cadmium distribution in the maize plant (Table 3; Figure 1) revealed that the environment was affected by the distribution to the grain, straw, and roots. There were no interactions between the sources of variation, with the exception of Cd distribution in the grain, which was affected by the environment × cultivar interaction (Table 3). 

The highest percentage of Cd distribution to the grain was attained in La Serena (3.0%) (*p* < 0.05), exhibiting significant differences between Chillán (0.8%) and Los Tilos (0.7%); however, there were non-significant differences between them (*p* > 0.05) (Figure 1a). The values for Cd distribution to the grain were 1.2%, 0.9%, and 0.9% for Cd rates 2, 1, and 0 mg·kg^−1^ CdCl_2_, respectively, with non-significant differences between them (*p* > 0.05) (Figure 1b). When comparing cultivars (Figure 1c), there was a higher Cd distribution to the grain in Pioneer (1.4%), while Syngenta and Dekalb obtained 0.9% and 0.8%, respectively, with non-significant differences between the three cultivars (*p* > 0.05).

Comparing environments for Cd distribution in the straw indicated that Los Tilos and Chillán did not show any significant differences one from the other (*p* > 0.05) and that their values were significantly higher than in La Serena (*p* < 0.05). The values for Cd distribution to the straw were 97.3%, 94.9%, and 72.5% in Los Tilos, Chillán, and La Serena, respectively (Figure 1a). The Cd rate and its effect on Cd distribution to the straw differed from that described for Cd distribution to the grain (Figure 1a,b); non-significant differences were detected (*p* > 0.05) with values of 90.8%, 90.2%, and 88.3% for rates of 2, 1, and 0 mg·kg^−1^ CdCl_2_, respectively (Figure 1b). Non-significant differences were observed between the Syngenta (90.4%), Pioneer (90.0%), and Dekalb (88.9%) cultivars (Figure 1c) for Cd distribution to the straw. 

Cadmium distribution to the roots when comparing environments (Figure 1a) revealed a higher value in La Serena (24.8%) (*p* < 0.05) than in the other environments. In turn, there were non-significant differences between Chillán and Los Tilos (*p* > 0.05) with values of 4.3% and 2.0%, respectively. As for Cd rates, values for distribution to the roots were 10.5%, 8.9%, and 8.3% for rates of 0, 1, and 2 mg·kg^−1^ CdCl_2_, respectively (Figure 1b); only the control was significantly higher than the other treatments (*p* < 0.05) with no differences between rates of 1 and 2 mg·kg^−1^ CdCl_2_ (*p* > 0.05). The comparison of cultivars (Figure 1c) indicated values for distribution to the roots of 10.3%, 8.7%, and 8.6% in Dekalb, Syngenta, and Pioneer, respectively (Figure 1c), with non-significant differences between them (*p* > 0.05). In addition, when comparing Cd distribution between the three maize plant structures, the straw usually concentrated the highest accumulation of the metal (*p* < 0.05), independently of the environment, Cd rate, and evaluated cultivar (Figure 1). The percentage accumulation of Cd in the grain was lower than in the root (*p* < 0.05) for the different Cd rates and in most of the environments and evaluated cultivars (Figure 1). 

Table 6 displays the translocation factors (TF) used to evaluate plant capacity to translocate Cd from the roots to the aerial part of the plant (straw + grain), which are the mean values (Table 6; *n* = 27) of the different: (a) environments; (b) Cd rates; and (c) maize cultivars. It is noted that there are significant differences in TF between environments (*p* < 0.05), TF in Los Tilos is 20 times higher than in Chillan and 2.6 times higher than in La Serena with values of 11.82, 4.63, and 0.57, respectively, for the three environments. Significant differences for TF between the different rates (*p* < 0.05) (Table 6) were observed for the 0, 1, and 2 mg·kg^−1^ CdCl_2_ treatments with values of 0.73, 1.96, and 2.84, respectively. The variations of TF, in accordance with the different Syngenta, Pioneer, and Dekalb cultivars (Table 6), did not indicate any significant differences between treatments (*p* > 0.05) with values of 2.31, 2.23, and 2.21, respectively. 

The BCF, or enrichment factor, is used to quantify plant capacity to accumulate Cd in the root with respect to Cd concentration in the soil. Table 6 indicates that BCF shows significant differences (*p* < 0.05) between environments and highlights the fact that La Serena has a greater capacity to accumulate Cd in the plant root than Chillán or Los Tilos, values are 1.2, 0.7, and 0.2, respectively. When comparing Cd rates, BCF showed non-significant differences among 0, 1, and 2 mg·kg^−1^ CdCl_2_ treatments (*p* > 0.05), and values were 0.66, 0.82, and 0.75, respectively. The same trend was observed when evaluating BCF for the different cultivars (Table 6) where non-significant differences were detected (*p* > 0.05) between Syngenta, Pioneer, and Dekalb with values of 0.68, 0.78, and 0.82, respectively.

On the other hand, BAF allows quantifying the capacity to accumulate Cd in the aerial part of the plant (straw + grain), with respect to Cd concentration in the soil. Table 6 specifies significant differences between environments for BAF (*p* < 0.05); a higher accumulation rate is observed in the Chillán environment, which is followed by Los Tilos (*p* < 0.05) and a lower bioaccumulation in La Serena (*p* < 0.05) with values of 2.54, 1.71, and 0.67, respectively. The same trend was observed when studying BAF in accordance with the Cd rates (Table 6); there were significant differences between 0, 1, and 2 mg·kg^−1^ CdCl_2_ (*p* < 0.05) and values were 1.7, 1.33, and 0.48, respectively. It should be noted that, when evaluating BAF for the different cultivars (Table 6), there were non-significant differences between them (*p* > 0.05) and the values were 1.35, 1.44, and 1.47 for Syngenta, Pioneer, and Dekalb, respectively.

Root TI (Table 6) exhibited non-significant differences between the environments in La Serena, Los Tilos, and Chillán and TI values were 0.15, 0.10, and 0.16, respectively (Table 6) (*p* > 0.05). Differences were neither observed for the different CdCl_2_ rates, and values were 0.17, 0.17, and 0.17 for 0, 1, and 2 g·kg^−1^ CdCl_2_, respectively (Table 6) (*p* > 0.05), nor for the three cultivars with values of 0.20, 0.14, and 0.16 in Syngenta, Pioneer, and Dekalb, respectively (Table 6) (*p* > 0.05). However, when evaluating TI in the grain, there were significant differences for both the environments and Cd rates (*p* < 0.05). When observing the environments, Los Tilos had the lowest value (*p* < 0.05) and was surpassed by Chillán, whereas La Serena had the highest value (*p* < 0.05). Values were 0.71, 1.29, and 1.87 in Los Tilos, Chillán, and La Serena, respectively (Table 6). Non-significant differences were detected between TI at the different Cd rates (*p* > 0.05) and values were 1.33, 1.24, and 1.30 for 0, 1, and 2 mg·kg^−1^ CdCl_2,_ respectively (Table 6). The same trend was observed for TI in the grain for the different cultivars, non-significant differences were observed (*p* > 0.05), and values were 1.32, 1.31, and 1.24 for Syngenta, Pioneer, and Dekalb, respectively (Table 6). Finally, TI of the straw exhibited the same trend as in the grain (Table 6) with significant differences only between environments (*p* < 0.05) where values were 1.78, 1.13, and 0.85 for La Serena, Chillán, and Los Tilos, respectively (Table 6). For the different CdCl_2_ rates (0, 1, and 2 mg·kg^−1^), TI values in the straw were 1.26, 1.25, and 1.25, respectively, and with non-significant differences (*p* > 0.05) (Table 6). The same statistical effect was obtained when comparing the different cultivars (*p* > 0.05), which had values of 1.33, 1.15, and 1.28 for Syngenta, Pioneer, and Dekalb, respectively (Table 6).

## 4. Discussion

The production of DM in the grain, straw, and root was not affected by the degree of anthropogenic contamination of the soil with Cd (1 and 2 mg·kg^−1^ CdCl_2_) or the different evaluated cultivars (Table 4); this is corroborated by TI (TI > 1) (Table 6) and concurs with Zhang et al. [34]. Other authors point out that soils contaminated with Cd exhibit decreased crop development that can fluctuate between 55% and 80% [35]; however, soil Cd contamination levels in that study were higher than those generated in the present study. Given that our results were similar to those obtained by other authors under similar Cd concentration conditions applied to the soil [13,36], it can be suggested that Cd concentrations achieved in the soil by applying CdCl_2_ are below the danger threshold of 3.5 mg·kg^−1^ [13]. However, the environment significantly affected DM production in Los Tilos (Table 4) for DM in the straw, grain, and root (Table 4). The lack of heat accumulation during the development stage and higher than optimal soil pH could be limiting factors for DM production, which concurs with other authors who point out a significant correlation between DM production and degree-day accumulation [36]. At the same time, the environment × cultivar interaction was affected by DM production, which corroborates what was previously mentioned and the fact that the response to the environmental factor is differentiating in accordance with the thermal and soil requirements of each cultivar [13,37].

Although initial total soil Cd was higher in La Serena compared with Los Tilos and Chillán (Table 1), this order was not maintained at the end of crop development (Table 2); this was probably generated by crop Cd absorption (Table 1 and Table 5) [29]. For total plant Cd extraction, non-significant differences were observed between environments (Table 5), which concur with Putwattana et al. [21]. However, mean total Cd extraction between different environments, Cd rates, and maize cultivars (22.4, 22.4, and 22.4, respectively) (Table 5) is higher than the mean among cultivars with phytoextraction potential reported by Slycken et al. [38] (18.5 g Cd·ha^−1^). These extraction levels demonstrate that the maize crop could be used for Cd phytoextraction [20,38] given that Cd extractions up to 42 g·ha^−1^ were reported in our experiment. On the other hand, Cd distribution in the different plant parts was affected by the environment with higher Cd absorption in the grain in La Serena (Table 5); this was 200% more than in Los Tilos, which, in turn, was 50% higher than in Chillán. This is mainly because DM production was higher in La Serena, which was pointed out by Trejo et al. [29]. These results concur with those obtained by Sarwar et al. [39], who also stated that high soil Zn concentrations, such as those found in the present study (Table 1), form Zn-phytochelatin complexes in the cell cytoplasm to substitute the Cd-phytochelatin complexes. This leaves Cd free inside the root cells and results in increased Cd translocation in the different plant parts, which was observed in the present study. However, low Cd extraction in the maize grain (Table 5) showed values within the range cited by several authors [13,40] with a mean Cd value of 0.18 g·ha^−1^ (mean of 27 values that considered three environments, three Cd rates, and three cultivars). This is an indicator of normal translocation for this species. Wahsha et al. [41] point out that Cd concentration in the maize grain was lower than the detection limit of 0.002 mg·kg^−1^, leading to low extraction levels for the maize grain, such as that observed in the present study. This could be due to several factors, for example, absorption and translocation limitations generated in the root [13], which suggests that maize plants have more efficient defense mechanisms than other crops to regulate Cd toxicity, including the accumulation of this metal at the root level [22]. Secondly, the available soil Zn concentration acts as an antagonist for plant Cd absorption [37]. Finally, the low Cd concentration could be attributable to high agronomic efficiency of nutrient use (kg of DM produced per kg of applied nutrient) obtained in the present experiment (Table 5), and which usually implies a Cd dilution effect [24]. 

It can also be observed in Table 5 that, for the environment, Cd rate, and cultivar, total Cd extraction in the straw is always significantly higher than in the roots or grain. These results concur with those reported by Liang et al. [42] and Stritsis et al. [43], who point out that Cd concentrations in maize straw are higher than in the rest of the plant. The same trend was observed in other crops, such as lettuce (*Lactuca sativa*) [44] and bread wheat (*Triticum aestivum*) straw [1]. Zhang et al. [45] explain these results by indicating that high S and Zn concentrations in the soil profile, also observed in the present study (Table 1), facilitate Cd translocation from the root to the aerial part of the plant. As previously mentioned, this higher Cd concentration in the straw indicates a higher translocation rate of this metal to the aerial part. Liang et al. [42] reported that it could be influenced by a decrease in sap flow that generates an increase of Cd concentration in the xylem when the crop is exposed to high Cd concentrations. It should be noted that the main factor for the higher translocation rate to the aerial part of the plant in the Los Tilos environment could be the high Ca levels in the soil profile associated with basic pH levels (Table 1). Sarwar et al. [39] pointed out that this generates increased Cd availability because it was substituted by Ca in the soil particles, thus increasing absorption by the plant and its translocation. Given the fact that high Cd extractions in the maize plant straw expose animals to this PTE and ultimately contaminate the food chain, which contributes in evaluating Cd levels in these maize cultivars destined for animal feed. Cadmium absorption by the maize plant is associated with the abovementioned factors and is benefited by high organic matter (OM) levels [12]; these levels were observed in the different evaluated environments in the present study and reached values close to 63 g·kg^−1^ in Chillán. 

As expected, the Cd concentration in the aerial part of the plant was proportional to the Cd concentration in the soil (Figure 1) where significant differences were noted in Cd extraction by the plant between the control and the 1 and 2 mg·kg^−1^ rates with more than 50% and 100% (Table 5), respectively. This concurs with the literature [46,47], which states that when the CdCl_2_ rate applied to the soil is higher, absorption rates of this PTE are higher. The same trend is observed when evaluating extraction levels in the same plant parts (grain, straw, and root) (Table 5). With respect to the studied maize cultivars, no effect on plant total Cd extraction was recorded and no differences existed between cultivars in the same plant part (Table 5); these results concur with Slycken et al. [38], who conducted a study with maize and pointed out that the different evaluated cultivars did not show any significant differences in Cd absorption. 

Plant capacity to absorb soil contaminants can be expressed as BCF, which indicates the relationship between the metal content in the plant tissue (root) and the soil [30,32,48]. Most of the BCF results in the present study fluctuated between 0.2 and 0.82, which concurs with results obtained by Usman and Mohamed [31], who pointed out that BCF values fluctuated between 0.38 and 0.9. In the different environment treatments, Cd rates, and cultivars, only the environment in La Serena reached values above the mentioned range (1.2) (Table 6). Different authors mention that BCF values >1 are high; this indicates that maize in the La Serena environment, independently of the cultivars and the degree of anthropogenic soil contamination, behaves as a plant with high Cd bioaccumulation efficiency at the root level [48]. On the other hand, low root BCF generally found in the de Los Tilos and Chillán environments can be explained by the maize plant’s capacity to prevent Cd absorption and probably for the low soil Cd bioavailability in accordance with previously-analyzed factors [22,37]. Furthermore, the different evaluated maize cultivars can be classified as excluding Cd because all the BCF values are <1 [31,48]. 

However, phytoextraction capacity has been expressed as TF and is defined as the relationship between the Cd concentration in the aerial part and the Cd concentration in the roots [36]. Results for TF obtained in the present study (Table 6) do not coincide with TF values recorded by other authors, who reveal that TF for different fertilization treatments, rotations, and degrees of Cd contamination were <0.5 [21,36]. Mean TF in the present study (3.26) is higher than the results reported by Liu et al. [36], which could be mainly influenced by soil pH in that study (pH between 8.31 and 9.06) and generate decreased Cd availability to the soil solution; this differs from the pH in the present study, which fluctuated between 5.74 and 8.25 (Table 1). The observed TF values in the present study (TF > 1) (Table 6) concur with results mentioned by other authors, who indicate TF values of 2.6 [33]. These results could classify these cultivars as plants with high Cd translocation efficiency from the roots to the aerial part [32]. The TF values obtained in Los Tilos (TF = 11.82) and BAF > 1 (Table 6) classify this environment as having the highest capacity to contaminate the food chain [7]. Therefore, more studies need to be conducted regarding PTE absorption and translocation to the aerial part of the crop in this environment, mainly cultivars used as a food sources for animals destined for human consumption. According to values reported by Usman et al. [32], translocation factors for cultivars, Cd rates, and environments in the present study exceeded the efficient plant classification limit of Cd translocation from the root to the aerial part, with the exception of the La Serena environment and the 0 mg·kg^−1^ Cd treatment rate where TF < 1. The three maize cultivars (Syngenta, Pioneer, and Dekalb) exhibited high risks of contaminating the food chain with Cd because of the high TF value (TF > 2) [32]. Based on these results, new maize cultivars should be made available or production management strategies be evaluated for this crop to reduce translocation rates of this metal to the aerial part of the plant, as well as more research to decrease both Cd absorption and accumulation in different maize plant tissues destined for animal consumption.

## 5. Conclusions

According to the results of the present study, it can be concluded that dry matter production is not affected by different degrees of cadmium (Cd) contamination generated in the soil of the different evaluated environments. The highest Cd concentration and extraction levels in the different evaluated maize cultivars were obtained in the aerial straw (stem + leaves), independently of the environment, Cd rates, and maize cultivars. 

Among the evaluated environments, the highest Cd concentration in the grain was observed in La Serena, but in no case was it higher than the established limits. According to the translocation factor (TF) (TF > 2) and bioaccumulation factor (BAF) (BAF > 1) values, the environments in Los Tilos and Chillán are classified as having a high capacity to contaminate the food chain for the evaluated cultivars and taking into account the degrees of Cd contamination generated in the soil. 

## Figures and Tables

**Table 1:** Soil physical and chemical properties (0–0.2 and 0.2–0.4 m depth).

Parameters	Environments and Depths (m)					
	La Serena		Los Tilos		Chillán	
	0–0.2	0.2–0.4	0–0.2	0.2–0.4	0–0.2	0.2–0.4
Clay (%)	20.2	20.3	21.5	27.3	20.7	15.9
Silt (%)	30.2	31.2	50.0	49.3	43.6	45.4
Sand (%)	49.6	48.5	28.5	23.4	35.7	38.7
Bulk density (g·cm^−3^)	1.76	1.80	1.20	1.24	1.00	1.05
pH (soil:water 1:2.5)	6.94	6.87	8.25	8.19	5.74	5.76
Organic matter (g·kg^−1^)	11.6	11.3	19.6	21.7	63.0	56.2
EC * (dS·m^−1^)	0.15	0.23	0.11	0.15	0.11	0.07
Available N (mg·kg^−1^)	18.0	20.0	11.0	14.0	40.0	38.0
Olsen P (mg·kg^−1^)	51.3	44.9	3.9	5.1	35.2	25.3
Exchangeable K (cmoL·kg^−1^)	0.85	0.67	0.35	0.41	0.65	0.39
Exchangeable Ca (cmoL·kg^−1^)	8.12	8.22	20.70	19.66	6.74	5.89
Exchangeable Mg (cmoL·kg^−1^)	2.41	2.61	0.92	0.86	0.95	0.72
Exchangeable Na (cmoL·kg^−1^)	0.59	0.69	0.49	0.40	0.16	0.19
Exchangeable Al (cmoL kg^−1^)	0.05	0.05	0.04	0.04	0.21	0.10
Available Fe (mg·kg^−1^)	21.5	20.8	16.7	17.0	59.8	46.5
Available Mn (mg·kg^−1^)	36.3	34.3	11.8	11.5	9.8	5.4
Available Zn (mg·kg^−1^)	4.5	4.4	1.1	1.2	0.7	0.6
Available Cu (mg·kg^−1^)	9.2	9.3	8.9	8.8	1.4	1.2
Available B (mg·kg^−1^)	2.3	2.4	0.8	0.8	0.5	0.4
Available S (mg·kg^−1^)	40.8	64.9	11.9	13.5	14.2	15.4
Total Cd (mg·kg^−1^)	1.33	1.49	0.52	0.51	0.21	0.18

* EC, electrical conductivity.

**Table 2:** Climatic characteristics of each environment during the season 2013–2014.

Parameters		La Serena			Los Tilos				Chillán
	Tm ^1^	pp ^2^	Ev ^3^	Tm	pp	Ev	Tm	pp	Ev
January	19.5	0.0	60.0	20.7	0.0	138.1	19.9	1.2	134.4
February	19.8	0.0	47.9	18.8	0.0	105.8	18.5	19.3	101.9
March	16.6	0.0	103.6	17.0	0.0	88.4	15.1	4.1	78.5
April	14.1	0.0	62.5	13.4	0.0	36.6	12.4	6.1	40.6
May	12.3	61.1	37.1	11.4	0.0	20.9	9.4	183.0	21.5
June	10.4	8.2	22.9	7.3	39.1	16.5	7.3	123.7	12.4
July	9.9	5.7	28.7	8.2	4.5	20.3	7.1	110.1	16.1
August	11.3	0.5	47.2	9.3	35.8	34.6	8.2	128.0	20.8
September	12.9	0.3	71.1	11.3	5.7	45.6	9.7	49.9	52.6
October	13.5	0.2	102.0	14.3	0.1	74.1	12.7	35.7	86.4
November	15.6	7.8	78.7	16.4	0.0	125.9	15.2	11.0	123.1
December	17.8	0.0	142.6	19.2	0.0	146.9	19.1	0.0	156.7
Total accumulate	-	83.8	804.3	-	85.2	853.7	-	672.1	845.0

**^1^** Tm, average temperature (°C); **^2^** pp, precipitation (mm); **^3^** Ev, evaporation (mm).

**Table 3:** Significance levels of the evaluated parameters for the experiments in three corn cultivars fertilized with three CdCl_2_ rates on three environments.

Parameter	L ^1^	R ^2^	C ^3^	L × R	L × C	R × C	L × R × C
Grain yield	**	NS	NS	NS	NS	*	NS
Straw DM production	**	NS	NS	NS	**	NS	NS
Roots DM production	**	NS	**	NS	**	NS	NS
Grain Cd uptake	**	**	NS	**	*	NS	NS
Straw Cd uptake	NS	**	NS	*	NS	NS	NS
Roots Cd uptake	**	**	NS	**	*	NS	NS
Total Cd uptake	NS	**	NS	NS	NS	NS	NS
Grain Cd distribution	**	NS	NS	NS	*	NS	NS
Straw Cd distribution	**	NS	NS	NS	NS	NS	NS
Roots Cd distribution	**	NS	NS	NS	NS	NS	NS

**^1^** L, Environments (La Serena, Los Tilos and Chillán); **^2^** R, Cd rates (0, 1 and 2 mg·kg^−1^); **^3^** C, Corn cultivars (Syngenta, Pionner and Dekalb); * Significant at *p* < 0.05; ** Significant at *p* < 0.01; NS, non-significant.

**Table 4:** Dry matter production (Mg·ha^−1^) according to maize plant parts for: (a) different study environments; (b) CdCl_2_ rates; and (c) maize cultivars.

Parameter Group	Parameters	Dry Matter Production (Mg·ha^−1^)			
		Grain	Straw	Root	Total
a	La Serena	15.45 a A	14.74 a A	2.04 a B	32.23 a
	Los Tilos	5.90 c A	7.04 c A	0.82 c B	13.76 c
	Chillán	10.67 b A	9.32 b A	1.36 b B	21.34 b
	Control	8.28 a A	9.78 a A	1.27 a B	19.33 a
b	1 mg·kg^−1^	7.68 a B	9.78 a A	1.25 a C	18.71 a
	2 mg·kg^−1^	8.06 a B	10.19 a A	1.29 a C	19.55 a
	Syngenta	8.19 a B	10.93 a A	1.48 a C	20.61 a
c	Pionner	8.12 a A	9.07 a A	1.10 c B	18.29 b
	Dekalb	7.71 a B	9.75 a A	1.23 b C	18.69 b

Different lowercase letters (a, b, c) in each column indicate significant differences compared with: (a) Environments; (b) Cd rates; and (c) maize cultivars according to Tukey’s test (*p* < 0.05). Different uppercase letters (A, B, C) between columns indicate significant differences between structures of the same plant for each: (a) environment; (b) Cd rate; and (c) maize cultivars according to Tukey’s test (*p* < 0.05).

**Table 5:** Cadmium extraction levels (g·ha^−1^) for each maize plant organ for: (a) different environments; (b) CdCl_2_ rates; and (c) maize cultivars.

Parameter Group	Parameters	Cadmium Extraction (g·ha^−1^)			
		Grain	Straw	Root	Total
a	La Serena	0.45 a C	18.87 b A	4.45 a B	23.77 a
	Los Tilos	0.08 c B	23.93 a A	0.24 b B	24.25 a
	Chillán	0.12 bc B	18.54 b A	0.61 b B	19.27 b
	Control	0.06 b C	2.24 c A	0.57 c B	2.87 c
b	1 mg·kg^−1^	0.19 a C	16.01 b A	1.53 b B	17.72 b
	2 mg·kg^−1^	0.25 a C	32.14 a C	2.06 a B	34.44 a
	Syngenta	0.16 a C	14.80 b A	1.43 a B	16.39 b
c	Pionner	0.19 a C	15.89 b A	1.07 a B	17.15 b
	Dekalb	0.14 a C	19.70 a A	1.66 a B	21.50 a

Different lowercase letters (a, b, c) in each column indicate significant differences compared with: (a) environments; (b) Cd rates; and (c) maize cultivars according to Tukey’s test (*p* < 0.05). Different uppercase letters (A, B, C) between columns indicate significant differences between structures of the same plant for each: (a) environment; (b) Cd rate; and (c) maize cultivar according to Tukey’s test (*p* < 0.05).

**Table 6:** Values for cadmium bioaccumulation factor (BAF), bioconcentration factor (BCF), translocation factor (TF), and tolerance index (TI) for grain, straw, and root according to different: (a) environments; (b) CdCl_2_ rates; and (c) maize cultivars.

Parameter Group	Parameters	BAF	BCF	TF	Tolerance Index		
					TI Grain	TI Straw	TI Root
a	La Serena	0.67 c	1.20 a	0.57 c	1.87 a A	1.78 a A	0.15 a B
	Los Tilos	1.71 b	0.20 c	11.82 a	0.71 c A	0.85 c A	0.10 b B
	Chillán	2.54 a	0.70 b	4.63 b	1.29 b A	1.13 b B	0.16 a C
	Control	0.48 c	0.66 c	0.73 c	1.33 a A	1.26 a A	0.17 a B
b	1 mg·kg^−1^	1.33 b	0.82 a	1.96 b	1.24 a A	1.25 a A	0.17 a B
	2 mg·kg^−1^	1.70 a	0.75 b	2.84 a	1.30 a A	1.25 a A	0.17 a B
	Syngenta	1.35 a	0.68 c	2.31 a	1.32 a A	1.33 a A	0.20 a B
c	Pionner	1.44 a	0.78 b	2.23 a	1.31 a A	1.15 a A	0.14 a B
	Dekalb	1.47 a	0.82 a	2.21 a	1.24 a A	1.28 a A	0.16 a B

Different lowercase letters (a, b, c) in each column indicate significant differences compared with: (a) environments; (b) Cd rates; and (c) maize cultivars according to Tukey’s test (*p* < 0.05). Different uppercase letters (A, B, C) between columns indicate significant differences between structures of the same plant for each: (a) environment; (b) Cd rate; and (c) maize cultivar according to Tukey’s test (*p* < 0.05).
